# A Review of Pickering Emulsions: Perspectives and Applications

**DOI:** 10.3390/ph15111413

**Published:** 2022-11-15

**Authors:** Fernanda Brito de Carvalho-Guimarães, Kamila Leal Correa, Tatiane Pereira de Souza, Jesus Rafael Rodríguez Amado, Roseane Maria Ribeiro-Costa, José Otávio Carréra Silva-Júnior

**Affiliations:** 1 Laboratory of Pharmaceutical and Cosmetic R&D, College of Pharmacy, Federal University of Pará, Belém 66075-110, Brazil; 2 Laboratory of Innovation and Development in Pharmaceutical Technology, Faculty of Pharmaceutical Sciences, Federal University of Amazonas, Manaus 69077-000, Brazil; 3 Laboratory of Pharmaceutical Technology, Faculty of Pharmacy, Food and Nutrition, Federal University of Mato-Grosso do Sul, Campo Grande 79070-900, Brazil; 4 Laboratory of Pharmaceutical Nanotechnology, College of Pharmacy, Federal University of Pará, Belém 66075-110, Brazil

**Keywords:** Pickering emulsion, solid particles, application

## Abstract

Pickering emulsions are systems composed of two immiscible fluids stabilized by organic or inorganic solid particles. These solid particles of certain dimensions (micro- or nano-particles), and desired wettability, have been shown to be an alternative to conventional emulsifiers. The use of biodegradable and biocompatible stabilizers of natural origin, such as clay minerals, presents a promising future for the development of Pickering emulsions and, with this, they deliver some advantages, especially in the area of biomedicine. In this review, the effects and characteristics of microparticles in the preparation and properties of Pickering emulsions are presented. The objective of this review is to provide a theoretical basis for a broader type of emulsion, in addition to reviewing the main aspects related to the mechanisms and applications to promote its stability. Through this review, we highlight the use of this type of emulsion and its excellent properties as permeability promoters of solid particles, providing ideal results for local drug delivery and use in Pickering emulsions.

## 1. Introduction

Emulsions are thermodynamically unstable systems in which droplets of an immiscible liquid are dispersed into another liquid with the help of a superficially active agent (emulsifying agent). In general, depending on the physicochemical characteristics of drugs, medications in liquid form lead to rapid absorption and greater bioavailability of the drug. Thus, drugs containing an insoluble active agent, in water or organic fluids, can be prepared in the form of a suspension and emulsion with good dispersion of liquid droplets, which enables its absorption and therapeutic action [1]. 

The preparation of these systems requires the use of emulsifying agents, which are necessary to maintain kinetic and thermodynamic stability for a longer period [2,3]. As emulsifying agents, surface-active substances can be used (Tween and Span), as well as polymer-forming mechanical barriers (polyethylene glycol (PEG) and polymethylmethacrylate (PMMA)). However, adverse effects, such as loss of moisture in the epidermis, skin atrophy, sensitivity to light, local irritation, and allergies, and hemolytic activity of pharmaceuticals and cosmetics in the form of emulsions have been reported due to the emulsifiers present in their formulations [4]. 

Some technological approaches have been taken to avoid the use of emulsifying agents. In this sense, the stabilization of an emulsion using only finely divided solid particles such as alginates, the so-called Pickering emulsions, is a promising alternative, since they improve the aspect of the formulation, provide good viscosity, and favor long-term stability [5]. These emerge as a good solution for the preparation of topical drugs and drug delivery to the skin, despite their thermodynamic instability that makes their development a complex challenge in pharmaceutical technology [6]. 

Pickering (1907) described that the presence of a layer of solid particles (proteins or other precipitated colloids) increased the lifespan of oil droplets and air bubbles in water [7]. Pickering emulsions generally provide a more stable system than classic emulsions obtained with a surfactant because they have advantages such as: reduced production cost, less adverse effects, low toxicity, and favorable biocompatibility [6,8,9]. 

Janus nanoparticles have been successfully used to produce Pickering emulsions, the way these oil/water interfaces can be more appropriated as a contrast to molecular surfactants. Janus nanoparticles are suitable for stabilizing Pickering emulsions as they offer more flexibility because one side of the particle can be kept very hydrophobic, while the polar side provides good stability and dispersibility in water. The polarity balance can be adjusted to match the polarity of the oil [10,11]. 

There is development of Pickering emulsions using carbohydrate polymers, and this class is dominated by two types of insoluble biopolymers that are widely abundant in nature: cellulose and starch. We also have examples of plant proteins, water-soluble albumins, salt-soluble globulins, alcohol-soluble prolamines, and acid/alkaline-soluble glutelins [12]. There are reports of studies that focused on solid particles that are commonly used as emulsifiers, such as clay, chitosan, and carbon nanotubes [9]. Ashby and Binks [13] found that Pickering emulsions can be stabilized by Laponite-type clay particles.

Corn zein is an example of a modern stabilizer and still occupies a large share of protein-based nanoparticles research [14]. Li et al. [15] showed that Pickering O/W emulsions were manufactured using zein/gum Arabic nanoparticles as stabilizers. Recently, activity for creating Pickering particles has expanded using prolamin-rich cereals such as sorghum, barley, and wheat, generating colloidal particles composed of kaphyrin, hodein, secalin, and gliadin [12].

The objective of this review article is to highlight the importance of Pickering emulsions and the use of solid particles to obtain them, with emphasis on clay minerals and their most current and impactful contributions to the pharmaceutical sciences. The physicochemical properties of these particles help in determining their functional possibilities, and it is expected to demonstrate that Pickering emulsions are potential candidates that can replace traditional emulsions and deserve the attention of industry and bioscience.

## 2. An Overview of the Physicochemical Characteristics of Pickering Emulsions

### 2.1. Formation of Pickering Emulsion

In the preparation of a Pickering emulsion, for the solid particles to be adsorbed at the oil–water interface, it is essential that the particles are fractionally wetted by the two phases. When solid particles are adsorbed, they increase the oil–water interfacial area and decrease energy, and thus reduce the driving force for particle transfer [16]. In summary, the formation of emulsions that are stabilized by finely divided particles depends on the wettability, concentration, size, density, and the form of packaging of the particles, the pH of the aqueous phase, and the presence of additives [17], which are the main analysis criteria for evaluating the stability of this type of emulsion.

We can cite examples of conditioning additives such as phosphates in Pickering emulsions, the selectivity of the catalytic system, and monitoring of cyclooctene, and the best were Na_2_HPO_4_ and [C_12_]H_2_PO_4_. The crystalline setting substitutes during microbial transformation in Pickering emulsions [18]. There are also studies with two types of interfacial additives: hydrophilic starch nanoparticles (HSNP) and hydrophobic starch nanoparticles (HOSNP) [19].

**Figure 1:**
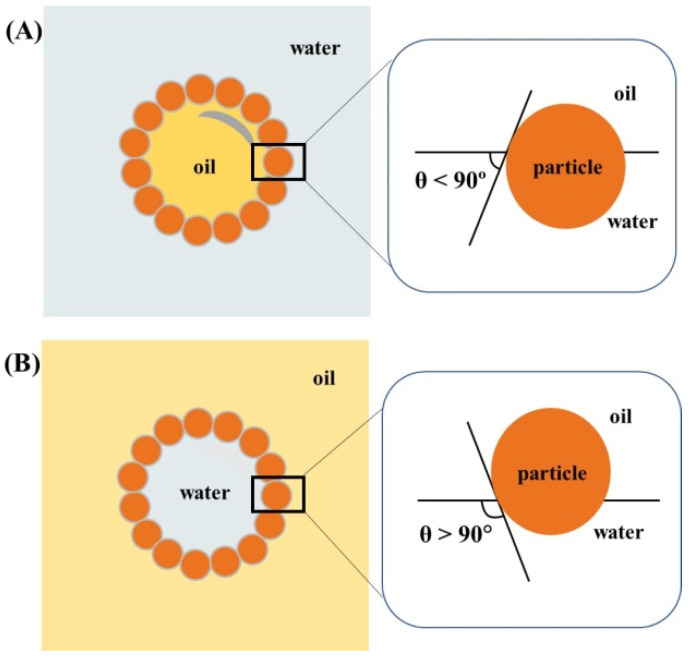
(**A**) Position of a solid particle at the drop interface with a contact angle of less than 90° corresponding to the formation of an O/W emulsion. (**B**) Position of a solid particle at the drop interface with a contact angle of greater than 90° corresponding to the formation of a W/O emulsion.

The wettability of solid particles used in Pickering emulsions is decisive to perform its function at the oil–water interface and can be quantified by measuring the contact angle between the particle and the interface. The type of emulsion (O/W or W/O) is determined by the wettability of the particles in the oil and water phases. The phase that wets the particle surface the most tends to be the continuous phase of the emulsion, with the contact angle being an important parameter (Figure 1). 

The particles that have a contact angle of less than 90° tend to form O/W emulsions due to their more hydrophilic character, and particles that have a contact angle of greater than 90° (hydrophobic particles) tend to form W/O emulsions. The results on the synthesis of polymer Janus particles revealed a strong contrast in hydrophilicity, with a measured contact angle of θ = 120° for the perfluorodecyl acrylate polymer (PFDA) and θ = 76° on the hexanediol diacrylate (HDDA) surface [20]. This reveals the affinity with the water or oil phases, which represents a preferential immersion of the emulsions (or good wettability, low contact angle) in one phase or another, which will eventually determine the curvature of the oil/water interface and the type of emulsion, with the contact angle being an important parameter [21]. The mechanism of obtaining Pickering emulsions includes the homogenization of the aqueous phase and the organic membrane (particles) [22]. The stabilization of these emulsions occurs due to the formation of a three-dimensional network composed of solid particles in the continuous phase. Within this network, the oily phase is retained, thus preventing the phenomenon of coalescence, sedimentation, or the formation of cream [23]. Denkov et al. [24] defended that Pickering emulsions break down if there are defects such as fractures or spaces in the layer of particles lining the oil droplets and, when there are breaks in the lattice of these particles, as effects we have thin films that separate droplets to break them apart, and they coalesce [25]. 

In classical emulsions, it is a surfactant molecule that reduces the interfacial energy to stabilize the emulsion; however, in Pickering emulsions, it is a layer of finely divided particles that reduces the tension and stabilizes the system. Therefore, in Pickering emulsions, factors such as stability, classification and characteristics (O/W or W/O), and morphology are highly dependent on the properties of the solid particles [9].

### 2.2. Particles Make the Difference: Their Properties and Stabilization

Nanoparticle-stabilized Pickering emulsions have gained great responsiveness due to their remarkable stability [26]. The potential advantage of nanoparticle-stabilized Pickering emulsions is the higher stability of the emulsion droplet in various experimental conditions, and that the emulsion can be simply demulsified after extraction processes, especially if the nanoparticles are magnetic [27].

Comparing the emulsions stabilized with particles with surfactant-stabilized emulsions, the former usually have different characteristics [28]. This type of emulsion is more stable for responding to stimuli due to the functional particles being anchored at the water/oil interface [5,9,29,30]. The control of stability and the type of Pickering emulsion play a significant role in these applications. 

Among the current methods for creating Pickering emulsions, the one that proposes adjusting the amphiphilicity of particles is comparatively effective and has attracted interest from the scientific community [31]. Honciuc cites that Janus particles have properties and functionalities due to the specific polarity contrast between two surface regions, and they resemble surfactants with a polar and a less polar side; therefore, Janus particles hold promise as “solid-state amphiphiles” [32].

Wu [10] corroborated that the amphiphilicity in Janus nanoparticles arising from a polarity contrast between two surface regions is well-recognized. A polarity inversion of these emulsions using polymers with different polarities can be observed and adopts preferential orientation within the monolayer interface at the oil/water interface. Thus, they showed that, in the absence of surfactants, the amphiphilicity of emulsions does not depend only on the size of the lobes, but also on the surface polarity contrast, which can be adjusted by altering the nature of the radical initiator.

When the stabilization of the Pickering emulsion is performed with micro- or nano-particles, a more robust barrier is formed, which prevents coalescence and maintains the stability of the emulsion for longer. Another important advantage of Pickering emulsions is that the absence of a surfactant results in low cytotoxicity and good biocompatibility [33]. In addition, due to the lower toxicity, controlled size, and high mechanical strength, polymerization via Pickering emulsion has been widely used [34]. 

The term “emulsion stability” typically refers to the ability of an emulsion to resist changes in its physicochemical properties over time [35]. This equilibrium refers to the ability of the emulsion to maintain the stability of its physical characteristics (size, shape, rheology, morphology, and others) for an extended period. Gravitational separation (sedimentation), coalescence, flocculation, and phase separation are mechanisms of instability that can be observed in emulsions. To determine the quality of these formulations, the following are evaluated: droplet size distribution, emulsion phase droplet morphology (optical microscope), wettability, rheology, shear stress, apparent viscosity, and stability, with respect to centrifugation [36].

## 3. Biomedical Applications of Pickering Emulsions

The applications of Pickering emulsions have attracted attention due to the benefit of the rapid development of particle synthesis techniques and the discovery of new colloids with adjustable surface properties, in addition to their constancy, their high payload capacity, and most importantly, the biocompatibility of particle stabilizers. Although surfactant-stabilized emulsions have been used successfully and extensively in various fields, they can cause adverse effects, such as irritation and hemolytic behavior [37,38]. 

The structural stability and biocompatibility of Pickering emulsions makes unalterable nanocellulose demonstrate potential for use in drug delivery and drug production. This type of emulsion offers remarkable capability for applications that need macroscopically homogeneous mixtures or efficient release systems, such as pharmaceuticals, cosmetics, food, fuel, and models for other materials, such as porous material, liquid foam and emulsion films, and aerospace core-shell nanofibers and hollow nanotubes [39]. Among the various applications of Pickering emulsions and their derivative materials, particle stabilizers can be endowed with certain special functionalities such as oxidation resistance, UV protection, environmental responsiveness, and even electromagnetic properties [38].

Regarding the experimental models that are used exclusively for the production of Pickering emulsions, a recent study stands out in which cellulose nanofibrils were used to stabilize an emulsion containing *Melaleuca alternifolia* oil, aiming at the production of antimicrobial cotton handkerchiefs. They were selected to simulate different contexts, in this case they have been used in the manufacture of antiseptic sheets considered in this global epidemiological context, as they are important due to the coronavirus pandemic. The coronavirus (SARS-CoV–2) pandemic stimulated the development of disinfection products, with handkerchiefs and cloths containing disinfectants being one of the products that increased consumption, mainly due to the ease of use of these products. In this work, cellulose was responsible for forming a layer that intensified the protection by collecting the oil droplets in the emulsions, through mechanical shear, which consequently provided greater stability and conservation [40]. 

Another important study containing natural stabilizers that deserves to be highlighted portrayed the development and characterization of Pickering emulsions stabilized by zein/chitosan particles. In this work, the encapsulation of vitamin D3 was carried out aiming at a formulation that presents antioxidant activities. These components enabled the protection of vitamin D3, as well as demonstrated that when forming emulsions, they provide a safe and stable vehicle for the delivery of bioactive compounds [41].

In addition to these studies, Pickering emulsions developed based on clay minerals were tested, where a study was developed in which silica nanoparticles and Fuller’s earth, composed of the clay mineral montmorillonite, were used to produce Pickering’s emulsions, with the clay mineral being used mainly because of its excellent dispersibility. The objective of this work was to obtain this emulsion to be used as a skin decontamination agent [42].

Wu et al. demonstrated a new approach to the manufacture of new non-spherical Pickering emulsion droplets using several different types of oil, which illustrated that the morphology and size of the droplets depend on the type of oil and the concentration of cyclodextrins. The cyclodextrins have also been used as emulsion stabilizers for topical formulations, and in addition, the results also confirmed greater stability and possible applications for the modified release of drugs, as well as attractive potential applications in the fields of drug release and food [43].

The excellent stability and biocompatibility of nanocellulose-stabilized Pickering emulsions offers great advantages in drug administration or food applications. Considering these characteristics, the applications of Pickering emulsions can be extended to cosmetics and paints, for example. These materials combine good mechanical properties and ecological characteristics. Therefore, they are of great interest in many fields, such as films for food packaging, separation/purification, and biomedical applications [39]. Protein-stabilized Pickering emulsions in the food industry have three main applications: expansive types of product formulation, encapsulation of bioactive components, and lipid protection [44].

With the progress of modern medicine, and since the vaccine is the most direct powerful tool to prevent epidemics transmitted by viruses, the adjuvant plays an important role in vaccine formulation. It is clear that good adjuvants can promote vaccine efficiency, and Pickering emulsions can be investigated as adjuvants [38]. Pioneering work on the use of Pickering emulsions in biomedical applications has also largely focused on drug encapsulation and delivery (such as topical and oral drug delivery) [45,46], bioimaging [47], and stimulus-responsive materials [43].

The application of Pickering emulsions for the encapsulation and delivery of bioactive ingredients, i.e., as drug emulsions, is still limited, and the pharmacokinetics of encapsulated substances using bioassays becomes necessary. Thus, to promote the bio-applications of Pickering emulsions stabilized by biocompatible particles, in-depth in vitro and in vivo studies are necessary [48].

## 4. Use of Apparatus for Obtaining Pickering Emulsions

The main processes for obtaining classic emulsions can also be used to produce Pickering emulsions [48]. Thus, by adding solid particles to the suspension, a Pickering emulsion can be prepared using emulsification techniques such as: high-speed homogenization with a rotor-stator, high-pressure homogenization, and sonication using an ultrasound processor [49,50]. In addition, some recent studies highlight new techniques of membrane emulsification and microchannel and microfluidic emulsification for obtaining Pickering emulsions [51,52,53]. 

### 4.1. Via a Rotor-Stator Mechanism

**Figure 2:**
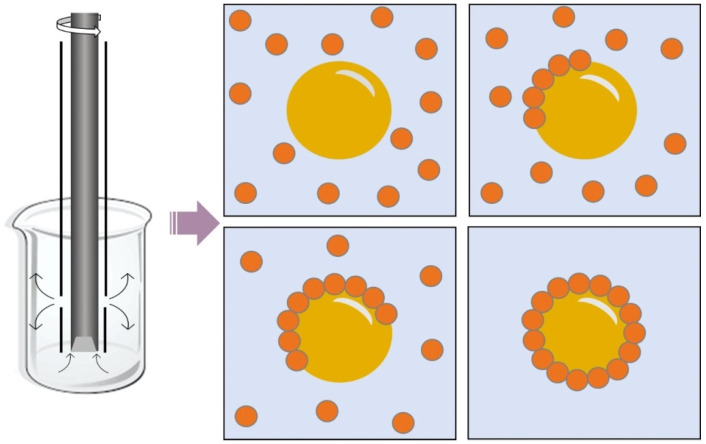
Schematic representation of the homogenization process using a rotor-stator and, as a result, the formation of a set of spherical particles adhered to the oil interface, which provides stability.

Regarding rotor-stator homogenizers (Figure 2), it is known that they are composed of an instrument with rotating blades that rotates around its own axis, known as the internal rotor, which rotates at high speed within a stationary outer sheath, the stator, which homogenizes the samples by means of mechanical tearing and with the aid of shear forces [54,55]. The functioning of this mixer is by the rotational movement of the blades, which causes the liquid sample to be dragged to one end of the mixture and expelled at high speed through the openings of the stator [51,55]. The differential speed that is close to the tolerance between the rotor-stator generates high levels of hydraulic cutting and promotes rapid homogenization and produces small droplets within the Pickering emulsions [56]. 

In general, the greater the shear applied in the emulsification process, the smaller the droplets produced, which increases the interfacial area, decreases the tension, and consequently, increases the stability. However, the high shear caused by excess energy can lead to coalescence of the droplets, thus affecting stability [55,56]. As an example of this mixer, there is the Ultra Turrax, which is responsible for producing turbulent flows that cause the disruption of the emulsion drops. The smallest droplet size that can be obtained is directly related to the geometry of the mixing head and the receptacle in which the mixture is created, and to the number of times it passes through the mixing zone, as well as to the stabilization system used in the study [57]. Thus, among the parameters that most influence the emulsion droplet size control, the rotation speed and homogenization time stand out [51]. 

Rotor-stator mixers have different advantages compared to other techniques for preparing Pickering emulsions, such as easy installation, ease of use and cleaning of the device, low investment costs, relatively high yield, and the speed of the process, which usually takes only a few minutes to obtain an emulsion [58]. There are advantages such as the small amount of liquid required, the ample distribution of droplet size obtained, and the high shear rate occurring between the rotor and the stator; in addition, these homogenizers permit the preparation of large volumes of emulsion [59,60].

The rotor-stator homogenizer ensures emulsification of different types of liquids and is therefore widely used in the preparation of cosmetic emulsions and other industrial products [55,56]. However, rotor-stators do have some disadvantages, such as the fact that most of them homogenize only a single sample at a time, although there are already some high-performance models and continuous models available on the market. In addition, despite helping to overcome the energy barrier, the high mechanical shear used in homogenization causes the breakdown of particle aggregates, which can result in a high polydispersity of emulsion droplets, and for this reason, the optimization of the process becomes necessary [43,57].

### 4.2. High-Pressure Homogenization

The high-pressure homogenization method is a continuous emulsification process most commonly used to prepare Pickering emulsions, and these emulsions can be prepared using this equipment, which contains a high-pressure valve which causes one of the liquids to transform and disperse uniformly in small globules inside the other [61]. High-pressure homogenization as one of the high-energy methods is an effective way to break the oil and water phases to generate tiny emulsion droplets. Studies have shown that the droplets formed by high-pressure homogenization are smaller compared to those formed by rotor-stator homogenization [51]. 

In addition to the effect of pressure, the greater the pressure exerted and the longer the homogenization time, there is an influence on the droplet size distribution, morphology, and droplet size [62]. High-pressure homogenization has advantages, such as the ability of the equipment to continuously produce and the possibility of obtaining small and uniform drops [63]. 

**Figure 3:**
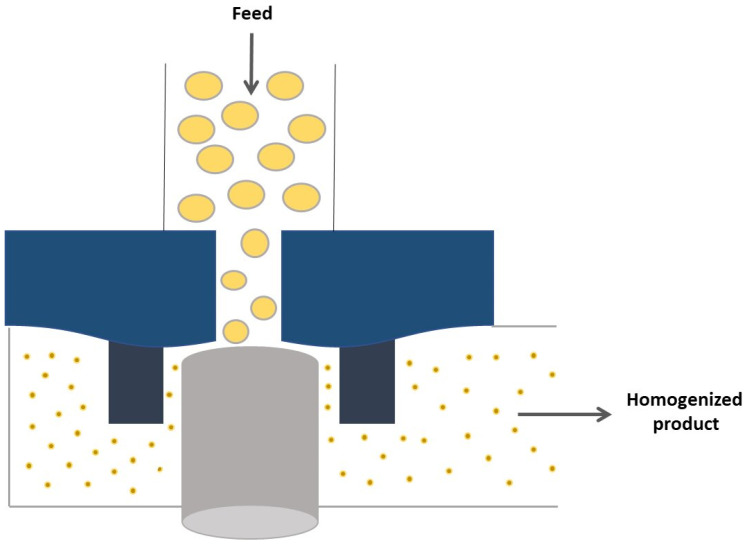
Operating scheme of a high-pressure homogenizer (adapted from Ref. [64]).

As a result, a pre-emulsion, which comprises a dispersion phase with large droplets, is forced through a high-shear region to promote turbulence and, therefore, aims to break down/disintegrate large droplets to generate a smaller particle size, as this equipment includes high-pressure pumps and homogeneous nozzles (Figure 3) [53]. 

In high-pressure homogenizers, the droplet size of the emulsion can be controlled during the emulsification process, both by the pressure value and by the number of homogenization cycles [65]. Homogenization conditions also play a key role in determining the size of the oil droplets in emulsions. In particular, the type of homogenizer used, the intensity of the disruptive forces generated, and the duration of homogenization are important factors. These types of intense disruptive forces can be produced using ultrasonic homogenizers or microfluidizers [66].

Although high-pressure homogenization is the most widely used in the continuous emulsification process in the industry, this technique is not predominant in the preparation of Pickering emulsions, probably because of its high cost and the risk of degradation during the process [51]. 

The resulting droplets are generally agglutinated and highly poly-dispersed [67] and, as a consequence, there is a lower reproducibility and a possible variation in the quality of the product emulsion per batch [53]. In addition, high-pressure homogenizers, normally used to produce emulsions on an industrial scale, consume a lot of energy, have a high maintenance cost, and are limited to producing only diluted emulsions of low viscosity, which generates the need for strict control of the conditions of preparation of Pickering emulsions using this apparatus [59].

### 4.3. Use of Ultrasound

**Figure 4:**
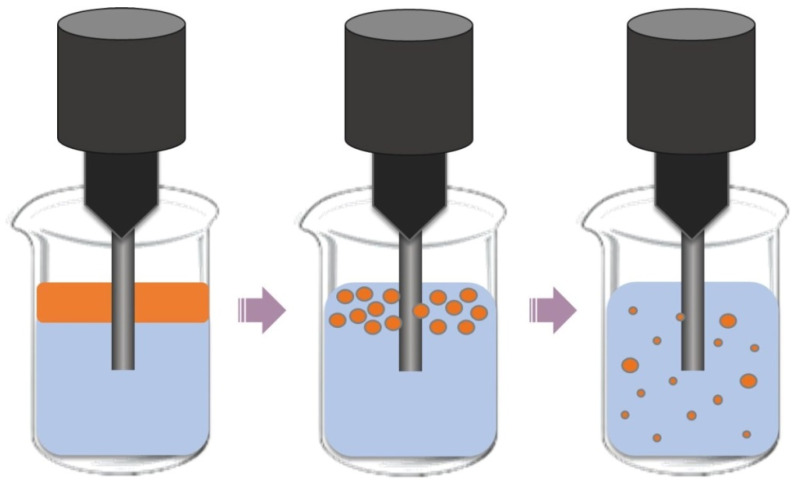
A schematic demonstration of the ultrasound device for emulsification.

Another method for obtaining Pickering emulsions is by using an ultrasonic processor. This equipment uses the phenomenon of cavitation, which causes the breakdown of droplets, and which can be defined as the rapid formation and collapse of bubbles in a liquid [57]. The ultrasound energy is concentrated near the probe, causing cavitation, and it can prepare an emulsion with smaller emulsion droplets (Figure 4) [68].

In the case of this method, only a small region of the fluid near the probe head is affected by the sonic waves, and the droplets present in the mixture being treated do not necessarily all experience the same amount of energy at the inlet. As such, this can be overcome up to a certain point by stirring of the emulsion at the same time as it is sonified. Studies show that one of the advantages of this method is that the droplet size of the emulsion decreases according to the sonification time [57]. 

Emulsions can be prepared by a series of mechanical processes, such as rotor-stator ultrasound treatment and high-pressure homogenization, to induce the mixture of oil and aqueous nanocellulose suspension, promoting adsorption of nanocelluloses at the oil/water interface to stabilize the emulsion [69]. 

The cavitation and shear stress caused by ultrasound can promote the adsorption of a stabilizer at the biphasic interface and, as a consequence, the droplet size of the emulsion will be smaller, and the stability will be greater [63]. A study concluded that the two-step emulsification approach involving shear and ultrasound was conducive to manufacturing high-stability emulsions [70].

## 5. Solid Particles Used as Stabilizing Agents

A wide range of particles can be used to stabilize Pickering emulsions. Nanoparticles and microparticles have advantages over larger particles, such as higher surface energy, composition, and architecture characteristics, and are an excellent choice for applications due to their customizable size and surface polarity, which enhances penetration into target tissue. The preparation and application of biodegradable nanoparticles have aroused interest in the research area and their application can be beneficial in the generation of Pickering emulsions. Pickering emulsions are stabilized by colloidal particles in the micro or nano range, including silica, clays, and carbon, as well as polymeric colloids such as starch, cellulose, and chitosan particles [71]. In this topic, we will point out, and then discuss, some of these particles used as stabilizing agents of Pickering emulsions, which are excellent alternatives to synthetic excipients that are used in the stabilization of pharmaceutical emulsions [72].

### 5.1. Polysaccharides in General

Polysaccharides are large natural polymers of high molecular weight that are formed by long chains of monosaccharides joined together by 1,4-glycosidic bonds [73]. The main advantages of these polymers are their biocompatibility, which is because they form part of biological membranes, in addition to their biodegradability, and because they do not present toxicity [74]. Cellulose-derived polysaccharides, such as hydroxypropylmethylcellulose (HPMC), hydroxypropylcellulose (HPC), and hydroxyethylcellulose (HEC), have great applications in the food and pharmaceutical industries as thickeners, stabilizers, and emulsifying agents [75]. 

Polysaccharides can stabilize an emulsion via different mechanisms. They can act by steric hindrance by forming an envelope that surrounds the oil droplets, thus avoiding the phenomenon of coalescence [76,77]. In addition, polysaccharides can cause an additional stabilizing effect in emulsions with hydrophobic groups or protein fractions, forming an adsorbed viscoelastic layer [78]. They also increase the viscosity of an emulsion when one is seeking to obtain the desired mechanical characteristics for the final product [79]. The efficiency of a stabilizing agent in an emulsion depends on the concentration of hydrocolloids in the aqueous phase and the characteristics of the structure formed by the biopolymer [79,80]. 

Among the polysaccharides most used in the stabilization of emulsions, cellulose, starch, and chitin and chitosan particles stand out, since they offer different advantages over synthetic particles [36]. The stability of emulsions using particles of biological origin is a valuable alternative for replacing synthetic stabilizers. Linked to this, the search for more sustainable alternatives provides new opportunities for obtaining this type of emulsion [81]. 

### 5.2. Cellulose and Derivatives

Cellulose is the most abundant biopolymer on Earth and the main reinforcing component in the plant cell wall [36]. It can be produced not only by plants, but also by fungi and some types of bacteria [82]. 

Cellulose consists of amorphous fibers and crystalline domains. Crystallinity and molecular weight depend on the origin of cellulose [83]. Just like starch, cellulose is odorless, tasteless, generally composed of non-toxic and non-irritating material, and widely used as a food, a pharmaceutical ingredient, and an excipient. As a pharmaceutical excipient, cellulose is used for oral or topical formulations and, to a lesser extent, for ophthalmic, injectable, or inhalable formulations [51]. 

This biopolymer can be divided into complex cellulose and pure cellulose, in which bacterial cellulose is produced especially by bacteria of the genus Gluconacetobacter, has a high degree of purity, high mechanical and tensile strength, and chemical and physical compatibility in association with other biopolymers [84,85,86]. Kalashnikov et al. demonstrated that bacterial cellulose fibrils can be used as particles to produce more stable Pickering emulsions. An emulsion is commonly considered more stable if it is resistant to physical changes over a practical period of time. It can be tested by various methods including centrifugation, filtration, agitation or agitation, low-intensity ultrasonic vibrations, or heating [87]. 

Paximada et al. pointed out that the stability mechanism in these emulsions can be explained by the flakes of the bacterial cellulose fibrils adsorbing the surface of the drop in the oil phase and forming a strong network that makes the emulsification of this type of cellulose greater when compared to other types of commercial cellulose [88].

Cellulose nanocrystals have been shown to be an efficient stabilizer of Pickering emulsions due to their sustainability, scalability, versatile fiber morphology, easy surface modification, good biocompatibility, and large surface area. This fiber is multifunctional and has a wide capacity for chemical modification and formation of versatile morphologies of semi-crystalline fibers [89]. Among the different solid stabilizers that exist, the use of micro-cellulose or nano-fibrillated cellulose may open-up new opportunities for the future of Pickering emulsions due to their size and rheological properties, due to their higher resistance ratios, and to their high degree of crystallinity [75], which should result in an improvement of the technique.

### 5.3. Starch

Cellulose, chitin, and starch, in that order, are the three most abundant polysaccharides in nature [90]. Starch is extracted from plants such as corn, potatoes, wheat, or rice. Starch is the most common carbohydrate in the human diet [91]. Powdered starch presents itself as discrete particles, called granules, of different sizes and shapes depending on its origin [92]. Starch is a white product, that is odorless, tasteless, non-toxic, and non-irritating. For these properties, it is used as a food ingredient and as an excipient in oral, solid pharmaceutical forms, and in semi-solid forms [93]. 

This polymer can be used as an excipient of Pickering emulsions and has the advantage of an amphiphilic character that favors its application in encapsulation and emulsification, as well as coalescence stability. According to some studies, this polymer does not demonstrate alterations in the rheological characteristics of emulsions over a long period [89,94].

### 5.4. Chitin and Chitosan

Chitin is an odorless, water-insoluble polysaccharide known as β-(1-4)-N-acetyl-D-glucosamine. It is extracted from the exoskeleton of crustaceans and shells, as well as being found in the cell wall of fungi. Its name chitin derives from the Greek word chiton, which means protective coating for invertebrates. Due to its versatility, it can be used as a flocculating agent or adsorbent in the preparation of Pickering emulsions. The main commercial sources of chitin are from shrimp, crab, and lobster waste [95].

Chitosan is the cationic polysaccharide and is scientifically known as (1-4)-2-amino-2-deoxy-β-D-glucan, which is obtained by deacetylation of chitin [96,97]. This deacetylation process can be carried out via hydrolysis using specific enzymes, or by chemical hydrolysis under alkaline conditions [98]. The main sources of commercial production of chitosan are the by-products of seafood processing, for example, crab and shrimp shells [99]. 

This polymer and its derivatives have a series of beneficial properties and have become a recurring theme in research on transdermal drug delivery, since it is non-toxic, biodegradable, biocompatible, immunologically neutral, antimicrobial, anti-inflammatory, and antitumor, with healing activity [1,99,100]. 

Sharkawy et al. used a chitosan emulsion as a carrier to deliver the trans-resveratrol, and the results of the skin penetration test showed that resveratrol had a high level of retention in the epidermis and dermis, which is of great importance for reducing the dosing time [101]. 

Asfour et al. [71] synthesized a drug containing rutin in a solubilized form, which was intended for wound healing through its loading in a new Pickering emulsion stabilized by self-aggregated chitosan particles, and they had a successful outcome. The authors reported that the emulsion showed long-term stability against coalescence of up to eight months, as well as greater rutin release efficiency compared to the simple drug suspension. In addition, the O/W formulation increased the wound healing rate when compared to the placebo formulation, which was proven by wound morphology, biochemical analysis, and histopathological examination.

Regarding its applications, it is known that chitosan is already used in cosmetic and pharmaceutical formulations, such as oral, nasal, parenteral, transdermal, ophthalmic, and implant administration [95]. Its solubility depends on its degree of deacetylation. For all these reasons, chitin and chitosan in their insoluble form are very attractive aspirants for pharmaceuticals, especially for Pickering emulsion, and are used for automatic particle aggregation [102]. The Table 1 contains the some complementary examples regarding solid particles used as stabilizing agents. 

### 5.5. Clay Minerals

Clay minerals, or simply clays, have been in use since ancient civilizations began [103]. The study of clays used in the medicinal arts is an ancient activity that is first documented in the writings of Greek and Roman philosophers: Aristotle (4th century BCE), Pliny the Elder and Dioscorides (1st century CE), and the Roman Galen of Pergamum (2nd century CE) [128]. In ancient Greece, clays were used as anti-inflammatory agents, because of their highly absorbent properties, and this custom endures to this day, and Dead Sea muds are used for cosmetic purposes, with some evidence of antibacterial properties arising from the large salt and sulfide content of the mud [129]. Table 2 contains the most used types of clays and their essential characteristics and their uses for applications in Pickering emulsions.

Due to the small particle size, absorbency, and plasticity, clays were first used for the treatment of wounds [154]. In recent decades, clays have been widely used in the formulation of solid and semi-solid dosage forms. The applications depend on the physical, chemical, mechanical, and rheological properties of the minerals, which act as an active component or as an excipient controlling the efficiency of the pharmaceutical forms and/or improving the bioavailability of the drug [155,156,157,158].

Clays have proven their effectiveness in both cosmetology and dermatology, have cleansing, moisturizing, soothing, regenerating, anti-inflammatory, sedative, antiseptic, and detoxifying effects, and they rejuvenate, tone, and nourish the skin [159,160]. Clays can suppress excess fat and toxins from the skin and have consequently been found to be effective for dermatological conditions such as furunculosis and ulcer treatment [161]. They are used to treat various skin conditions such as seborrheic dermatitis, psoriasis, chronic eczema, and acne [158,162,163,164,165]. 

The study by Carretero et al. reports that clay minerals can be powerful in cosmetic products, especially for hair care, due to their peculiar properties. Particular properties such as lubricants, carriers, inert bases, viscosifying agents, stabilizers for suspensions and emulsions, as protection against environmental agents, for skin adhesion, and as fat adsorption agents are specifically attributed to its capacity as a multitasking system. It also includes high specificity in surface areas, high absorption capacity, favorable rheological characteristics, chemical inertness, and low or zero toxicity [161]. An overview of the use of clay in cosmetics for skin and hair is reported, presenting a general introduction regarding its minerals and their wide potential for application in the biomedical field, which may be useful for the formulation of new solid shampoo formulas [166]. 

Recent research shows that clay minerals can protect against ultraviolet (UV) light due to their high specific surface area, thus providing effective coverage of the skin’s surface. At the same time, the addition of the clay fraction would increase the sun protection factor (SPF) of the product, decreasing the amount of synthetic UV filters required to obtain a certain SPF value [167].

Constantly, small amounts of particles varying in size from silt to clay are associated with secondary kaolinite, particularly quartz, illite, and anatase, but also smectite, hematite, goethite, pyrite, and marcasite, while, in primary kaolin deposits, significant amounts of quartz, feldspars, muscovite, tourmaline, zircon, rutile, and pseudo-rutile are common [168,169]. With the exception of Antarctica, noticeable kaolinite occurrences are recorded on all continents. The most notable kaolinite mining districts worldwide are allocated in the United States, Uzbekistan, Brazil (eastern Amazon region), the United Kingdom, Germany, Czech Republic, Republic of Korea, Ukraine, and Bulgaria, as well as other minor kaolinite occurrences in different locations in other European, American, and Asian countries.

To ensure the suitability of kaolin minerals as pharmaceutical-grade products, they must meet certain requirements specified in the major pharmacopoeias (European, British, American, and Japanese) regarding mineralogy, color, organic impurities, adsorption power, swelling power, substances that are soluble in mineral acids, loss by ignition, chemical composition (mainly chlorides, sulfates, iron, calcium, and heavy metals), and microbial contamination. Manuals of pharmaceutical excipients cite other technical properties that kaolins must have, including particle size distribution being expressed as of average size, dynamic viscosity, whiteness, acidity or alkalinity, refractive index, equilibrium moisture content, and specific gravity [170,171,172].

Pharmaceutical grades of kaolin are in high demand for use as an excipient in formulations of solid and semi-solid dosage forms, including tablets, capsules, pellets, granules, powders, pastes, poultices, ointments, creams, lotions, and suspensions. In critical chemical purity requirements and limits of acceptance of physical and chemical criteria, various concentrations of kaolin are prescribed for many drug forms to perform certain functions in pharmaceutical manufacturing processes. The most important functionalities of kaolin used as an excipient are as a diluent, binder, and disintegrant, and in pelleting and granulation, amorphization, particle film coating, emulsification, and suspending agents [171,173].

Silva et al. observed an exceptional performance of kaolinite particles in mixtures of Brazil nut oil and water, which they attributed to the presence of two separate domains, resembling a Janus particle. They observed that shortly after mixing water and Brazil nut oil, kaolinite was able to orient itself to form a water-in-oil (W/O) emulsion [142].

### 5.6. Other Types of Solid Particles for Use in Pickering Emulsions

The widely variable characteristics of these particles allow their use in a wide range of applications, and the interfacial assembly of these particles in this type of emulsion offer a new opportunity for further exploration of their performance [43]. Synthetic polymers are also widely used in the development of pharmaceutical formulations due to the improvement of their functional properties, and it is important to highlight that hybrid materials facilitate the stages of absorption and distribution of drugs in different organisms [33,174,175]. 

Xu et al. [176] produced active edible films based on gelatin and obtained excellent results by incorporating a Pickering emulsion loaded with dihydromyricetin (DMY) stabilized by dialdehyde cellulose (DAC) in the gelatin matrix.

As important candidates for stabilization of Pickering emulsions, we can cite polylactic acid (PLA), polyglycolic acid (PLGA) and its derivative polymers, and polycaprolactone (PCL), with the exception of the importance of testing the compatibility of these polymers with the other components of the formulations, in which the oily phase of the emulsion is not always compatible with these stabilizers [51]. In addition, there are still polymers that are not biodegradable, but that can be used as emulsion stabilizers, and these are poly(N-isopropylacrylamide) (PNIPAm) and poly(ethylene oxide) (PEO) [176].

Another synthetic product widely used in Pickering emulsions is silica, mainly due to this emulsion presenting high stability in the presence of inorganic particles. Silicon dioxide (SiO_2_) or silica, as it is commonly known, is a mineral oxide that constitutes one of the essential components of the earth’s crust, in addition to the oxides of magnesium, aluminum, calcium, and iron. In addition, this material has good resistance to acidic and basic environments, and another advantage of silica is the control of the size and structure of the formulation obtained [4].

Pena-Muniz et al. [177] used hydrophilic silica powder (Aerosil^®^200) as a stabilizing agent for Pickering emulsions containing *Bertholletia excelsa* HBK oil. Ribeiro-Costa et al. [178] developed and patented a product and its topical use in the form of Pickering emulsion from Pracaxi oil (*Pentaclethra macroloba*) containing vitamin E (tocopherol acetate) in water and stabilized using hydrophilic silica. 

In 1988, Levine et al. [179] successfully fabricated an oil-in-water (O/W) emulsion stabilized using hydrophilic silica nanoparticles with the diameter of approximately 572 nm and found that a monolayer of silica nanoparticles was formed at the oil–water interface. Jiang et al. [180] synthesized monodispersed silica nanoparticles with different diameters (50, 130, and 230 nm) and then chemically modified the silica nanoparticles to make them hydrophobic to stabilize water-in-oil (W/O) Pickering emulsions.

Xu et al. [176] manufactured CaCO_3_ microparticles in the presence of xanthan gum and poly(sodium 4-styrenesulfonate) using the Pickering emulsion mold method and in situ gelation and, as a result, showed that the core-shell structure can regulate the controlled release behavior of the trapped functional component and that it has potential in the delivery system.

## 6. Conclusions

The understanding of the relationships between composition, properties, and physicochemical aspects of solid particles in the preparation of Pickering emulsions is still very restricted. Pickering emulsions enhance new approaches to the development of pharmaceutical and even food products, as it is a new and promising strategy for functional interface technology. 

In this paper, we presented the use of Pickering emulsions for bioactive delivery using mainly natural ingredients. It is a good tool for loading these bioactives with different physicochemical characteristics, using vegetable oils as well. Within the universe of natural ingredients, clay minerals have a series of benefits and characteristics that make them unique in relation to conventional solid particles. 

Thus, one of the most important points in the study of Pickering emulsions is the need to have particles that are compatible with their purpose and understand their behavior in stabilizing the emulsions produced. This type of formulation has long-term stability, is environmentally friendly, and has a controlled and targeted release of active substances. Pharmaceutical forms using Pickering emulsion molds present a wide range of potential applications in the fields of cosmetics, food, and biomedicine.

This review has provided a general description of the influence and notoriety in the selection of solid agents and contains a collection of information on the main polymers used, as well as their exceptional characteristics. In addition, it was possible to determine the importance in the development potential of Pickering emulsions, since there is a predominance of clay minerals in Brazilian soils where they are abundant. It is important to apply advanced delivery systems to natural products that are traditionally used. 

The authors believe that emulsified systems, specifically Pickering emulsions, for drug delivery would improve the therapeutic efficacy of traditional medicines, primarily using natural compounds. This review concisely discussed the advances in research of biocompatible particles for the stabilization of Pickering emulsions. 

## Figures and Tables

**Table 1:** Some complementary examples regarding solid particles used as stabilizing agents.

Stabilizing Agents	Chemical Composition	Applications	Particle Size	Occurrences	Ref.
Cellulose	(C_6_H_10_O_5_)n	Cellulose is used in the food, biomedical, and pharmaceutical areas, as a drug and cosmetic delivery system, also used in wound healing and dressing membranes. Cellulose nanocrystals can be used as drug carriers in the pharmaceutical industry, paper industry, food industry, and support matrix for catalysts.	Lower than 38 μm	It is produced by plants, fungi, and some types of bacteria.	[103,104,105,106,107,108,109,110,111,112]
Starch	(C_6_H_10_O_5_)_n_	It is used for delivery systems of pharmaceutical ingredients and bioactive food ingredients, enzyme inhibitor, and adsorption agent.	>49.8 µm	Starch is found in seeds, roots, tubers, and bulbs of vegetables.	[113,114,115,116,117]
Chitin and Chitosan	(C_8_H_13_O_5_N)n and C_56_H_103_N_9_O_39_	The potential for multidimensional application, ranging from applications in the food field such as nutrition, biotechnology, materials science, drugs and pharmaceuticals, agriculture and environmental protection, and gene therapy as well.	Approximately 6.9 μm	Chitin is the most commonly found aminopolysaccharide in nature, being part of the exoskeleton of crustaceans, insects, and the cell walls of fungi, and chitosan is included in the enzymatic or chemical deacetylation of chitin.	[118,119,120,121]
Polylactic acid and polyglycolic acid	C_5_H_8_O_5_	Bioplastics become attractive materials for biological and medical applications, can be used for implants and other surgical applications such as sutures, and in regenerative surgery implants.	Approximately 125 µm	Polyester obtained from the polymerization of lactic acid, produced by fermentation or chemical synthesis	[122,123]
Silica	SiO_2_	Biomedical applications have the potential to be used in the diagnosis and therapy of many diseases.	In between 28 and 500 nm	The volcanic and sedimentary origin silica occurrences	[124,125,126,127]

**Table 2:** Some complementary examples regarding clay minerals for use in Pickering emulsions.

Classes/Species	Chemical Composition	Applications	Size Variations	Locations or Occurrences	Model Applications	Ref.
Montmorillonite Smectites (Bentonites and vermiculites)	(Na,Ca)_0.33_(Al,Mg)_2_Si_4_O_10_ (OH)_2_·(H_2_O)n	In cosmetology (formulations of make-up, shampoos, and skin emulsions) and in drug delivery, and in the synthesis of polymer–clay nanocomposites in tissue engineering.	Average diameter of around 7 µm. They are alkaline compounds in nature	Paraíba (Municipalities of Cubati and Boa Vista) northeast Brazil	Montmorillonite-stabilized Pickering emulsions were formed at high salinities.	[130,131,132,133,134]
Illites (Glauconites)	(Si_4_)(Al,Mg, Fe)_2,3_ O_10_(OH)_2_.(K,H_2_O)	Has been widely used as an adsorbent in water treatment applications in removing different metal ions and dyes.	Ranges from 125 to 500 µm	Usually occur in microscopic crystals on land masses	-	[135,136,137]
Kaolinite (Kaolin)	Al_4_Si_4_O_10_(OH)_8_	Application as an oil/water emulsion stabilizer and oil recovery and has been used for centuries in pharmaceutical preparations of intestinal adsorbent drugs and other therapeutically useful applications.	Particle sizes of 2–5 µm	In the state of Rio de Janeiro, there are currently occurrences of kaolin in the municipalities of Magé, Valença, Sapucaia, Petrópolis, Itatiaia, Araruama, and Rio de Janeiro. Capim River in Pará	Study aimed to investigate the preparation and characterization of oil-in-water (O/W) Pickering emulsions stabilized with three different phyllosilicates: kaolin, halloysite, and palygorskite. Stable O/W emulsions could be obtained without additional surfactant or surface treatment.	[137,138,139,140,141,142,143]
Chlorite	(Mg,Fe)_3_(Si,Al)_4_O_10_(OH)_2_·(Mg, Fe)_3_(OH)_6_	Widely used in the pharmaceutical industry as lubricants, desiccants, disintegrants, diluents, binders, pigments, and opacifiers.	Approximately 0.5 to 10 µm and thickness ranging from 0.1 to 1.0 µm	They are common in clayey rocks, recent marine sediments, most soils, and in mines. Found in Furnas and Lageado, in the state of São Paulo, Panelas, and Canoas, in the state of Paraná and Rio Grande do Sul	-	[137,144,145,146]
Laponite	Na_0,7_[(Si_8_MgLi_0,3_)O_20_(OH)_4_]_0,7_	Medicine, pharmacy, and food packaging; in addition, presents great potential as an adsorbent of organic pollutant compounds such as pesticides. Used in the polymerization of Pickering emulsions.	1 and 30 nm diameter	Synthetic material with a similar structure and composition to natural hectorite.	Pickering emulsion polymerization using laponite clay as a stabilizer to prepare armored “soft” polymer latexes	[147,148,149,150,151,152,153]

## Data Availability

Data sharing not applicable.
